# Construction of a high density linkage map and genome dissection of bruchid resistance in zombi pea (*Vigna vexillata* (L.) A. Rich)

**DOI:** 10.1038/s41598-019-48239-5

**Published:** 2019-08-12

**Authors:** Kitiya Amkul, Lixia Wang, Prakit Somta, Suhua Wang, Xuzhen Cheng

**Affiliations:** 1 Department of Agronomy, Faculty of Agriculture at Kamphaeng Saen, Kasetsart University, Kamphaeng Saen Campus, Nakhon Pathom, 73140 Thailand; 2 Center of Advanced Studies for Agriculture and Food, Kasetsart University Institute of Advanced Studies, Kasetsart University, Bangkok, 10900 Thailand; 3 Center of Excellence on Agricultural Biotechnology: (AG-BIO/PERDO-CHE), Bangkok, 10900 Thailand; 4 Institute of Crop Sciences, Chinese Academy of Agricultural Sciences, Beijing, 100081 China

**Keywords:** Agricultural genetics, Plant breeding

## Abstract

Zombi pea (*Vigna vexillata*) is a legume crop that is resistant to several biotic and abiotic stresses. *Callosobruchus maculatus* and *Callosobruchus chinensis* are serious stored-insect pests of legume crops. We constructed a high-density linkage map and performed quantitative trait loci (QTLs) mapping for resistance to these insect species in zombi pea. An F_2_ population of 198 individuals from a cross between ‘TVNu 240’ (resistant) and ‘TVNu 1623’ (susceptible) varieties was used to construct a linkage map of 6,529 single nucleotide polymorphism markers generated from sequencing amplified fragments of specific loci. The map comprised 11 linkage groups, spanning 1,740.9 cM, with an average of 593.5 markers per linkage group and an average distance of 0.27 cM between markers. High levels of micro-synteny between *V*. *vexillata* and cowpea (*Vigna unguiculata*), mungbean (*Vigna radiata*), azuki bean (*Vigna angularis*) and common bean (*Phaseolus vulgaris*) were found. One major and three minor QTLs for *C*. *chinensis* resistance and one major and one minor QTLs for *C*. *maculatus* resistance were identified. The major QTLs for resistance to *C*. *chinensis* and *C*. *maculatus* appeared to be the same locus. The linkage map developed in this study will facilitate the identification of useful genes/QTLs in zombi pea.

## Introduction

The genus *Vigna* is an important plant taxon containing nine cultivated legume crops, including mungbean [*Vigna radiata* (L.) Wilczek], blackgram [*Vigna mungo* (L.) Hepper], cowpea [*Vigna unguiculata* (L.) Walp.], rice bean [*Vigna umbellata* (Thunb.) Ohwi and Ohashi], Bambara groundnut [*Vigna subterranea* (L.) Verdc.], azuki bean [*Vigna angularis* (Ohwi) Ohwi and Ohashi], moth bean [*Vigna aconitifolia* (Jacq.) Maréchal], zombi pea [*Vigna vexillata* (L.) A. Rich] and creole bean (*Vigna reflexo-pilosa* Hayata) which are grown in various cropping systems on over 25 million hectares in Asia, Africa, Australia and America^1,2^. These *Vigna* crops are generally susceptible to biotic and abiotic stresses^1^. Among the *Vigna* species, zombi pea (*Vigna vexillata*) is of particular interest. It is a pan-tropical herbaceous legume plant. Wild zombi pea is distributed widely throughout tropical and sub-tropical regions of Africa, Asia, Australia and America^3^. The species often develops storage roots that are edible and consumed by people in Africa, India, Australia and Southeast Asia^4–6^. Edible tubers of *V*. *vexillata* have a ~15% protein content, which is much greater than the contents of potato, yam and cassava^7^. Moreover, the yield of *V*. *vexillata* tuberous roots can be as high as 7 tons per hectare^8^. The cultivated form of *V*. *vexillata* is found in a limited area of Africa and Asia (Bali, Indonesia) in which both seeds and tuberous roots are consumed^2^.

Owing to its wide distribution, *V*. *vexillata* has adapted to several climatic and environmental conditions, such as infertile^6^, alkaline^9^, acidic^10^, and saline soil^11^, as well as drought^11^ and waterlogging^12^. In addition, it is resistant or tolerant to several insect pests and their related diseases, such as *Callosobruchus maculatus*^13^, *Zabrotes subfasciatus*^13^, *Maruca testulalis*^14^, *Maruca vitrata*^15^, *Clavigralla tomentosicollis*^14^, and cowpea mottle carmovirus^16^. The successful hybridization between zombi pea and cowpea, to transfer the former’s genetic resistance genes to pests and diseases, has been reported^17^.

Bruchids or seed weevils (Coleoptera: Bruchidae) are a group of insects that destroy the seeds of legume crops. Bruchid infestations of *Vigna* crops initially occur in fields where female bruchids lay eggs on young pods. The larvae bore through the pods to the seeds. Here, the larvae grow and develop into adults by consuming the seed’s nutrients. After harvest, the adult bruchids emerge from the seeds and start new infestations by laying eggs directly on stored seeds, which can result in a total loss of a seed lot within 2–4 months^18^. Although chemical fumigation can be used to control bruchid infestations, the use of resistant cultivar(s) is the best management strategy^18^. In general, seeds of cultivated *Vigna* species are susceptible to bruchids, especially the cowpea weevil (*C*. *maculatus* L.) and azuki bean weevil (*C*. *chinensis* F.)^18–20^. These pests are widely distributed in nearly all continents owing to international seed trading. However, in general, seeds of both cultivated and wild *V*. *vexillata* are completely resistant to the *C*. *maculatus* and *Z*. *subfasciatus*, and this resistance results from the presence of the non-protein amino acid para-aminophenylalanine (PAPA) in the seeds^13^.

Although *V*. *vexillata* has the potential to be a source of resistance genes to biotic and abiotic stresses for plant breeding and to be a new feed crop in the future, little is known of its genetics and limited genomic resources are available. Therefore, it is necessary to increase our knowledge and develop genomic tools to exploit the genetic potential of *V*. *vexillata* for use in breeding *Vigna* and other crops. Here, our objectives were to (i) develop a high-density linkage map for *V*. *vexillata* and (ii) identify quantitative trait loci (QTL) for seed resistance to *C*. *chinensis* and *C*. *maculatus* in *V*. *vexillata*.

## Results

### Specific Locus Amplified Fragment Sequencing (SLAF-seq) data and genotyping

After pre-processing, 187.10 Gb of raw data, containing 733.52 M reads, were generated. On average, the Q30 (quality scores of at least 30, indicating a 1% chance of error, and thus 99% confidence) was 94.05%, and the GC content was 36.63%. The numbers of reads for cultivated zombi pea ‘TVNu240’ (resistant) and wild zombi pea ‘TVNu 1623’ (susceptible) were 23,183,922 and 24,285,995, respectively. The total read number for offspring was 3,464,884. After read clustering, 4,044,822 single nucleotide polymorphisms (SNPs) were detected of which 2,202,256 (55.45%) were polymorphic. After filtering the SNP markers lacking the parent information and showing low read depths, 378,433 polymorphic markers were successfully genotyped and grouped into eight segregation patterns, ab × cd, ef × eg, hk × hk, lm × ll, nn × np, aa × bb, ab × cc, and cc × ab. In total, there were 233,975 SNP markers showing the segregation pattern of aa × bb.

### Characteristics of the genetic map

**Figure 1:**
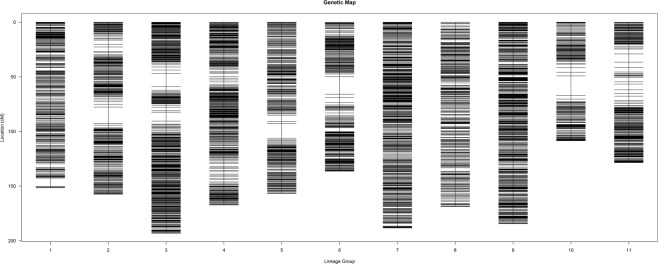
A high-density genetic linkage map constructed for the *Vigna vexillata* F_2_ population of a cross between ‘TVNu 240’ and ‘TVNu1623’ using 6,529 SNP markers from the SLAF sequencing.

After a series of screenings, 6,954 SNP markers were used for a genetic linkage analysis. Among these markers, 6,529 (93.89%) were successfully clustered into a linkage map of 11 linkage groups (LGs) (Fig. 1), indicating the relatively high quality of the genetic map. Table 1 summarises the characteristics of the linkage map constructed in this study. The map was 1,740.8 cM in length, with an average distance of 0.27 cM between adjacent markers. The numbers of markers per LG ranged from 267 on LG10 to 1,080 on LG3, with an average of 593.5 markers. The shortest LG was 108.52 cM (LG10), and the longest LG was 193.13 cM (LG3) (Table 1; Fig. 1). The average length of a LG was 158.25 cM. Large gap(s) (gap between two adjacent markers >10 cM) existed on LGs 2, 3, 5, 6, and 10.

**Table 1:** Features of the high-density genetic linkage map constructed for the *Vigna vexillata* F_2_ population of a cross between ‘TVNu 240’ and ‘TVNu1623’ using SNP markers generated by SLAF sequencing.

Linkage group	No. of Markers	Length (cM)	Average distance between markers (cM)	Maximum gap (cM)
1	546	151.6	0.28	7.58
2	494	157.4	0.32	14.84
3	1,080	193.1	0.18	11.81
4	643	167.3	0.26	3.05
5	440	156.7	0.36	13.74
6	470	136.3	0.29	16.03
7	773	188.3	0.24	2.43
8	517	168.7	0.33	3.09
9	882	184.4	0.21	2.32
10	267	108.5	0.41	17.88
11	417	128.5	0.31	7.52
**Total**	**6,529**	**1,740.8**	**0.27**	**17.88**

### Comparative genome analysis

**Figure 2:**
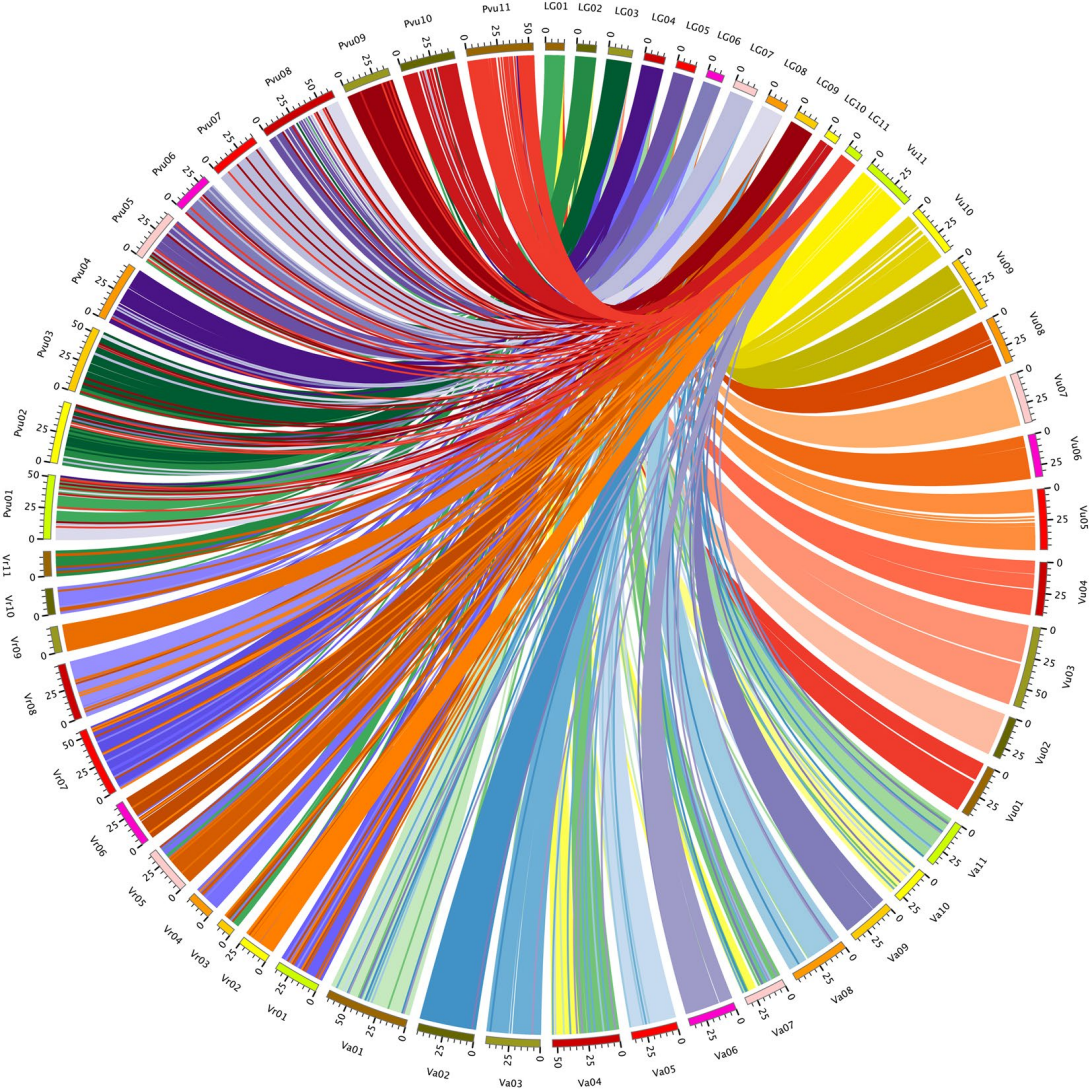
Circos map showing genome synteny between the genetic linkage map of *Vigna vexillata* (LG01–11) and the reference genome sequences of cowpea (*V*. *unguiculata*; Vu01–11), mungbean (*V*. *radiata*; Vr01–11), azuki bean (*V*. *angualaris*; Va01–11), and common bean (*Phaseolus vulgaris*; Pvu01–11).

The genome comparison revealed the presence of highly conserved microsyntenic blocks between zombi pea and cowpea, mungbean, azuki bean, and common bean (Fig. 2; Supplementary Table S1). The zombi pea genome showed the greatest synteny with the cowpea genome. Zombi pea LGs 1, 2, 3, 4, 5, 6, 7, 8, 9, 10, and 11 corresponded to cowpea chromosomes 1, 2, 3, 4, 5, 6, 7, 8, 9, 10, and 11, respectively, to mungbean chromosomes 2/3/5, 11, 7, 1, 4/5, 10, 8, 6, 5, 9, and 2, respectively, to azuki bean chromosomes 4/7, 10, 1, 11, 4/7, 5, 8, 3, 2, 9, and 6, respectively, and to common bean chromosomes 1/5, 2/3, 2/3, 4, 5/8, 6, 7, 1/8, 9, 10, and 11, respectively.

### Variation in resistance to *C*. *maculatus* and *C*. *chinensis* in the parents and F_2_ population

The zombi pea varieties TVNu 240 and TVNu 1623 differed in their responses to *C*. *chinensis* infestation, with 0% and 48.67%, respectively, of seeds being damaged. The area under the disease progress stairs (AUDPS), which indicates the progression of infestation severity, was 0 in TVNu 240 and 705 in TVNu 1623. In the F_2_ population the level of damage caused by *C*. *chinensis* varied from 0 to 96%, with an average of 11.60%, and the AUDPS varied from 0 to 1,970, with an average of 190.61.

In addition, TVNu 240 and TVNu 1623 had very different responses to *C*. *maculatus* infestation, with 0% and 94%, respectively, of seeds being damaged. The AUDPS was 0 in TVNu 240 and was 2,115 in TVNu 1623. In the F_2_ population, the level of damage caused by the bruchids varied from 0% to 98%, with an average of 26.99%, and the AUDPS varied from 0 to 2,193.88, with an average of 552.52.

**Figure 3:**
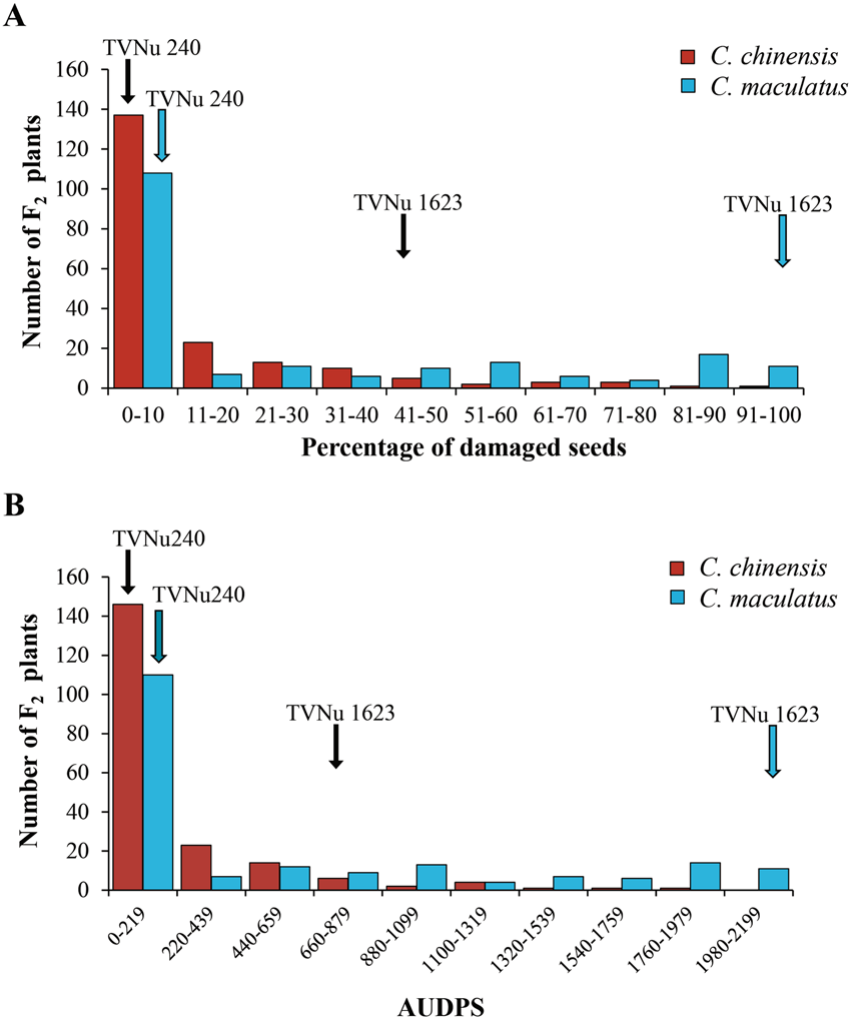
Frequency distributions of the percentage of damaged seeds (**A**) and the area under the disease progress strairs (AUDPS) (**B**) resulting from *C*. *chinensis* and *C*. *maculatus* infestation in the *Vigna vexillata* F_2_ population of a cross between ‘TVNu 240’ and ‘TVNu1623’.

The frequency distributions of percentage of seeds damaged and the AUDPS as a result of *C*. *chinensis* and *C*. *maculatus* infestations in the F_2_ population were continuous but highly skewed towards the resistant TVNu 240 (Fig. 3a,b). Thus, resistance to *C*. *chinensis* and *C*. *maculatus* in TVNu 240 appears to be a quantitative trait and may be controlled by several genes.

The correlation between the percentage of damaged seeds and AUDPS value in each bruchid species was positive and very high, at 0.99 (*P* < 0.001) in both species (Table S2). Correlations between traits of the two bruchid species were positive and moderately high, at 0.70 to 0.72 (*P* < 0.001) (Table S2). Thus, resistance to *C*. *chinensis* and *C*. *maculatus* appears to be controlled by some common genetic factors.

### Heritability

Broad-sense heritability (*H*^2^) estimates for the percentage of seeds damaged by *C*. *chinensis* and *C*. *maculatus* were 57.55% and 98.44%, respectively. The *H*^2^ estimates for AUDPS resulting from *C*. *chinensis* and *C*. *maculatus* infestations were 87.701 and 98.24, respectively. These indicated that resistance to *C*. *chinensis* and *C*. *maculatus* was principally controlled by genetic factors.

### QTL analysis

**Figure 4:**
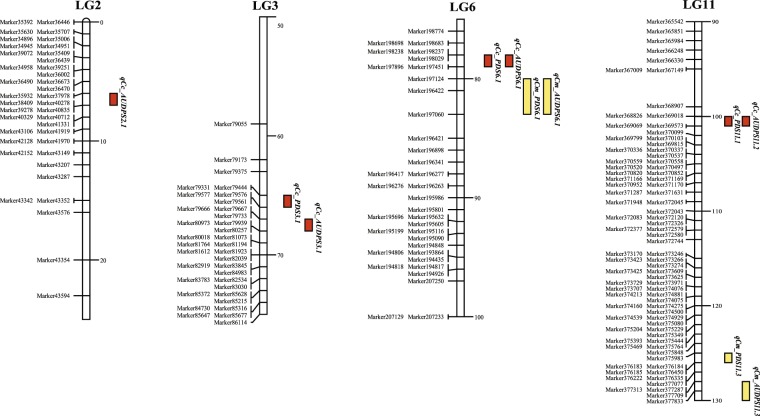
Linkage group locations of the QTLs identified for the percentage of damaged seeds and the AUDPS resulting from *C*. *chinensis* and *C*. *maculatus* infestations in the *Vigna vexillata* F_2_ population of a cross between ‘TVNu 240’ and ‘TVNu1623’. Bars next to linkages indicate locations of the QTLs based on 1-LOD support. Only partial linkages at QTL sites are shown.

For *C*. *chinensis*, three (one major and two minor) QTLs on LGs 3, 6, and 11 were identified for percentage of damaged seeds (Table 2; Fig. 4). The major QTL, *qCc_PDS6*.*1*, was at 78 cM, between Marker79444 and Marker79577, on LG6 and explained 25.51% of the total trait variation (PVE) in the F_2_ population. The two minor QTLs each accounted for less than 10% of PVE in the population. At these QTLs, alleles from TVNu 240 decreased the percentage of damaged seeds. One major and three minor QTLs each on LGs 2, 3, 6, and 11 were identified for AUDPS (Table 2; Fig. 4). The major QTL, *qCc_AUDPS6*.*1*, was at 78 cM on LG6 and explained 23.99% of the PVE in the F_2_ population. All the minor QTLs showed PVE values less than 10%. At all the QTLs detected for AUDPS, alleles from TVNu 240 increased AUDPS (increased the resistance). The QTLs for the percentage of damaged seeds and AUDPS identified on the same LG were located in the same region (Table 2; Fig. 4).

**Table 2:** QTLs associated with seed resistance to *Callosobruchus chinensis* and *Callosobruchus maculatus* detected in the F_2_ population of the cross between cultivated zombi pea ‘TVNu240’ and wild zombi pea ‘TVNu1623’.

Bruchid species	Trait^a^	LG^b^	QTL name	Position^c^	LOD score	PVE^d^ (%)	Add^e^	Dom^f^
*C*. *chinensis*	PDS	3	*qCc_PDS3*.*1*	65.4	4.80	8.33	0.15	−9.38
		6	*qCc_PDS6*.*1*	78.0	14.12	27.14	11.72	−0.94
		11	*qCc_PDS11*.*1*	99.6	4.60	7.86	6.17	−3.44
	AUDPS	2	*qCc_AUDPS2*.*1*	6.0	4.75	7.65	82.45	−105.17
		3	*qCc_AUDPS3*.*1*	66.8	4.94	8.42	14.79	−161.10
		6	*qCc_AUDPS6*.*1*	78.0	12.77	23.76	191.50	−1.26
		11	*qCc_AUDPS11*.*1*	99.6	4.86	8.15	107.99	−65.41
*C*. *maculatus*	PDS	6	*qCm_PDS6*.*1*	80.6	33.28	52.41	27.33	−20.26
		11	*qCm_PDS11*.*1*	125.6	6.61	7.21	11.38	−6.70
	AUDPS	6	*qCm_AUDPS6*.*1*	80.0	30.74	51.82	444.63	−430.01
		11	*qCm_AUDPS11*.*1*	128.4	4.84	5.66	195.12	−156.34

^a^PDS = percentage of damaged seeds, AUDPS = area under the disease progress stair.

^b^linkage group.

^c^Position (centimorgan) on the linkage map.

^d^Phenotypic variance explained by the QTL.

^e^Additive effect.

^f^Dominant effect.

In case of *C*. *maculatus*, one major and one minor QTLs on LGs 6 and 11 were found for the percentage of damaged seeds (Table 2; Fig. 4). The major QTL, *qCm_PDS6*.*1*, was at 81 cM, between Marker197124 and Marker196422, on LG6 and explained 52.41% of the PVE. The minor QTL, *qCm_PDS11*.*1*, was located at 125 cM, between Marker375983 and Marker376183, and explained 7.21% of the PVE. At these two QTLs, alleles from TVNu 240 decreased the percentage of damaged seeds. Similarly, one major and one minor QTLs on LGs 2 and 11 were identified for AUDPS (Table 2; Fig. 4). The major QTL, *qCm_AUDPS6*.*1*, was at the same position as *qCm_PDS6*.*1* and explained 52.46% of the PVE. The minor QTL, *qCm_AUDPS11*.*1*, was at 67 cM, between Marker360510 and Marker360570, on LG11 and explained 6.26% of the PVE. At both *qCm_AUDPS6*.*1* and *qCm_AUDPS11*.*1*, alleles from TVNu 240 increased the AUDPS. The major QTLs for resistance to *C*. *maculatus* and *C*. *chinensis* were located in the same genomic region.

## Discussion

In recent years, underutilised legume crops, including zombi pea, have been praised as potential sources of genes for crop improvement and food security in the face of climate change and future population increases^21^. Zombi pea is resistant to several biotic and abiotic stresses. Gene discovery in zombi pea can be useful for crop improvement. However, the lack of genomic resources and breeding tools in zombi pea hinders gene discovery in this species. Next-generation sequencing technologies are highly useful for genomics study of underutilised crops^21^. Genetic linkage mapping is important in genomics studies aimed at crop improvement because they facilitate gene mapping and association analyses needed for gene discovery^22^. In this study, we successfully constructed a high-density linkage map for zombi pea that will be used to identify genomic regions associated with bruchid resistance.

### Linkage map of zombi pea

Previously, there were only three linkage maps for zombi pea, with the first linkage map of this species being developed 13 years ago^23–25^. The map developed by Marubodee *et al*.^24^ had the greatest number of markers and density. It was composed of 559 markers (84 simple sequence repeats and 475 SNPs based on restriction site-associated DNA-tagged sequences) markers, with a mean distance of 1.8 cM between adjacent markers, which grouped into 11 LGs. In our present study, by applying the SLAF-seq technique and the reference genome sequence of cowpea, the linkage map developed for zombi pea was substantially improved, containing 6,529 SNP markers that resolved into 11 LGs, with a mean distance of 0.27 cM between adjacent markers. Compared with the high-density linkage maps developed for mungbean^26^, azuki bean^27^, and cowpea^28^, our linkage map contained a greater number of markers than the mungbean (1,321 markers) and azuki bean (2,032 markers) maps but less than the cowpea map (17,739 markers). The high-density linkage map developed in this study is useful for genomics studies of zombi pea.

### Genome synteny with related legume species

Previous comparative genome analyses among species belonging to the genus *Vigna*, including zombi pea, cowpea, mungbean, and azuki bean, based on common markers of linkage maps have revealed extensive macro-syntenic relationships^24,25^. In this study, the syntenic maps based on sequence comparisons revealed highly extensive micro-synteny levels among zombi pea and other *Vigna* species, as well as common bean. All these species have the same number of chromosomes (2n = 2x = 22). Previous studies showed high genome conservation between *Vigna* species and common bean^29–31^. As expected, a synteny analysis revealed that zombi pea had the greatest level of genome conservation with cowpea compared with other legume species. Zombi pea and cowpea both originated from Africa and share several common morphological characteristics^32^. Although zombi pea and cowpea are classified into different subgenera (*Plectrotropis* and *Vigna*, respectively) of the genus *Vigna*, successful hybridization between the two species has been reported^18^. Owing to the high level of genome conservation (Fig. 2), available reference genome sequences of cowpea^30^ can be used for genomic analysis and gene identification in zombi pea.

### Genetics of bruchid resistance in zombi pea

In general, zombi pea is completely resistant to *C*. *maculatus*^14,33^; therefore, it is not possible to investigate the genetics of the resistance. To investigate the genetics of the bruchid resistance in zombi pea, a large set of zombi pea germplasm (>400 accessions) from several countries were screened against *C*. *maculatus* and *C*. *chinensis* (P. Somta, unpublished data) to identify susceptible zombi pea varieties. Most of the germplasms were completely resistant to both bruchid species, but some accessions were moderately or highly susceptible to the bruchids. As a result, TVNu 240 and TVNu 1623 were used as resistant and susceptible parents, respectively, in this study.

A continuous distribution but with high levels of skewedness towards the resistant parent for the percentage of damaged seeds, resulting from both *C*. *chinensis* and *C*. *maculatus* infestations, were observed in the F_2_ population (Fig. 3a,b). This suggested that the resistance is quantitative, and may be controlled by one or a few major locus/loci with dominance over the susceptible loci coupled with some modifying loci. In *Vigna* species, both quantitative and qualitative loci control resistance to *C*. *chinensis* and/or *C*. *maculatus*. The quantitative resistance against bruchids has been reported in rice bean^34,35^, black gram^36^, and *Vigna nepalensis* Tateishi and Mexted^37^, while qualitative resistance has been reported in mungbean (single dominant gene)^38^, black gram (two duplicate genes)^39^, and cowpea (two recessive genes)^40^. Nonetheless, the high heritability estimated for the bruchid resistance (~90%) in *V*. *vexillata* suggests that the resistance in this species is mainly the result of genetic factor(s).

### QTLs for bruchid resistance in zombi pea

The QTL analysis identified one major and three minor QTLs for *C*. *chinensis* resistance and one major and two minor QTLs for *C*. *maculatus* resistance in zombi pea accession TVNu 240 (Table 2; Fig. 4). Among these QTLs, the major QTLs *qCc_PDS6*.*1* and *qCc_AUDPS6*.*1* (that conferred resistance to *C*. *chinensis*), as well as the major QTLs *qCc_PDS6*.*1* and *qCc_AUDPS6*.*1* (that conferred resistance to *C*. *maculatus*), were mapped to a similar position. These QTLs are very likely the same locus. Thus, we considered them the same QTL, named *qBr6*.*1*. Similarly, the minor QTLs *qCc_PDS11*.*1* and *qCc_AUDPS11*.*1* that conferred resistance to *C*. *chinensis* were mapped to the same position. Thus, we considered them the same QTL, named *qBr11*.*1*. In addition, the minor QTLs *qCm_PDS11*.*1* and *qCm_AUDPS11*.*1* that conferred resistance to *C*. *maculatus* were mapped to a similar positions. Again, they were considered the same QTL, named *qBr11*.*2*. *qBr11*.*1* and *qBr11*.*2* was ~30 cM apart.

Among *Vigna* species, bruchid resistance has been well studied in mungbean^41,42^ in which a major QTL, *qBr5*.*1*, for resistance to *C*. *chinensis* and *C*. *maculatus*, is located on chromosome 5 and a gene encoding a polygalacturonase inhibitor is a candidate resistance gene. The markers associated with the *qBr6*.*1* for bruchid resistance in zombi pea, Marker197124 and Marker196422, are on mungbean chromosome 10. Therefore, *qBr6*.*1* in zombie pea and *qBr5*.*1* in mungbean are not the same locus.

Although Birch *et al*.^14^ reported that PAPA in seeds of zombi pea acts as a defence chemical against *C*. *maculatus* and *Z*. *subfasciatus*, Bresson^43^ showed that some zombi pea accessions, differing in resistance to *C*. *maculatus*, do not have significantly different amounts of PAPA in their mature seeds. This suggested that PAPA may not be the principal defence chemical against *C*. *maculatus* and *Z*. *subfasciatus* in zombi pea. Lattanzio *et al*.^44^ demonstrated that, in general, seeds of zombi pea resistant to *C*. *maculatus* contain high contents of an α-amylase inhibitor. The α-amylase inhibitor from common bean is an antibiosis that negatively affects the growth and development of *C*. *chinensis*, *C*. *maculatus*, and *Z*. *subfasciatus*^45,46^. Genome comparisons revealed that Marker197124 and Marker196422 flanking *qBr6*.*1*, which confers resistance to *C*. *chinensis* and *C*. *maculatus* in zombi pea, were on common bean chromosome 6 at the positions 18.726 Mb and 19.329 Mb, respectively. Three genes *Phvul*.*006G087700* (Chr06: 19,974,245–19,976,281), *Phvul*.*006G200500* (Chr06: 29,738,762–29,740,823), and *Phvul*.*006G200800* (Chr06: 29,750,251–29,752,428), each coding for α-amylase inhibitors, are on common bean chromosome 6. *Phvul*.*006G087700* is ~650 Kb away from Marker196422 and can be considered a candidate gene for bruchid resistance in zombi pea. Further studies are necessary to confirm the association between this gene and resistance in zombi pea.

## Materials and Methods

### Plant materials

An F_2_ population of 198 individuals derived from the cross ‘TVNu 240’ × ‘TVNu 1623’ was used in this study. TVNu 240 is a cultivated zombi pea (*V*. *vexillata* var. *macrosperma*) from the Central African Republic and is completely resistant to *C*. *maculatus* and *C*. *chinensis*, while TVNu 1623 is a wild zombi pea from Nigeria and is highly susceptible to *C*. *maculatus* and moderately susceptible to *C*. *chinensis*. The seeds of these two accessions were obtained from the International Institute of Tropical Agriculture, Ibadan, Oyo State, Nigeria.

The F_2_ population and 10 plants of each parent were grown under field conditions using 0.75 × 0.75 m spacing at Kasetsart University, Kamphaeng Sean Campus, Nakhon Pathom, Thailand from December 2017 to March 2018. Genomic DNA was extracted from young leaves of each F_2_ and parental plant using the CTAB method^47^. Mature pods and seeds of each plant were harvested separately for bruchid evaluation.

### SLAF-seq marker analysis

The genomic DNAs extracted from the parental lines and from each of the 198 F_2_ plants were used for SLAF library construction and sequencing following Sun *et al*.^48^, with minor modifications. Briefly, a pre-experiment SLAF-seq analysis was performed using the reference genome of cowpea^30^ to obtain the optimal restriction enzymes, which would avoid repetitive SLAFs, and an even SLAF distribution. Then, a SLAF library was prepared based on the results of the pre-experiment analysis. Genomic DNA from each entry was digested at 37 °C with *Hin*CII and *Rsa*I (NEB, Ipswich, MA, USA), incubated with the Klenow Fragment (3′ → 5′exonuclease) (NEB) and dATP at 37 °C to add single-nucleotide A overhangs to the digested fragments, and the A-tailed DNA fragments were then ligated to Duplex Tag-labelled Sequencing adapters (PAGE purified, Life Technologies) using T4 DNA ligase. PCR was performed using diluted restriction-ligation DNA samples, dNTPs, Q5® High-Fidelity DNA Polymerase and PCR primers (Forward primer: 5′-AATGATACGGCGACCACCGA-3′, reverse primer: 5′-CAAGCAGAAGACGGCATACG-3′) (PAGE-purified, Life Technologies). PCR products were then purified using Agencourt AMPure XP beads (Beckman Coulter, High Wycombe, UK) and pooled. Pooled samples were separated using 2% agarose gel electrophoresis. Fragments ranging from sizes 314 to 464 bp (with indexes and adaptors) were excised and purified using a QIAquick gel extraction kit (Qiagen, Hilden, Germany). Gel-purified products were diluted. Paired-end sequencing (each end being 125 bp) was performed using an Illumina HiSeq X-ten system (Illumina, Inc; San Diego, CA, USA) in accordance with the manufacturer’s recommendations at the Biomarker Technologies Corporation (Beijing, China).

### Sequence data grouping and genotyping

Reads generated from the sequencing were compared to the cowpea reference genome sequence using BWA software. SLAF marker identification and genotyping were performed as per by Sun *et al*.^48^. Low-quality reads (quality score <20e) were eliminated and the raw reads were assigned to 198 F_2_ individual samples using the duplex barcode sequences. After trimming the barcodes and the terminal 5-bp positions from each high-quality read, the clean reads were clustered together based on their sequence identities. Sequences that mapped to the same locus with over 90% identity levels were defined as one SLAF locus. SNP loci between the two parents were detected in which SLAFs with >3 SNPs were removed. Alleles of each SLAF were defined based on parental reads with sequence depths >10-fold and offspring reads with sequence depths >2-fold. In a diploid species, such as zombi pea, one marker locus can contain a maximum of four genotypes; thus, SLAF loci with >4 alleles were discarded. Nonetheless, only SLAFs with 2, 3, or 4 alleles were considered potential markers. Because *V*. *vexillata* is a self-pollinating species, only polymorphic markers showing homozygous states between the parents were selected. Genotype scoring was conducted using a Bayesian approach as described by Sun *et al*.^48^. Subsequently, three processes were performed to screen for high-quality markers. First, markers with average sequence depths less than fourfold in the parents were discarded. Second, markers having >25% missing data were removed. Third, markers with significant segregation distortion (*P* < 0.001) were initially excluded from genetic map construction and then added later as accessory markers.

### Genetic map construction

Marker loci were partitioned primarily into LGs based on their locations on the cowpea reference genome. Next, the modified logarithm of odds (MLOD) scores between markers was calculated to further confirm the robustness of the markers for each LG. Markers with MLOD scores < 5 were filtered prior to ordering. HighMap strategy^49^ was used to order the SNP markers and correct genotyping errors within LGs. First, recombinant frequencies and LOD scores were calculated using a two-point analysis to infer linkage phases. Then, enhanced Gibbs sampling, spatial sampling and simulated annealing algorithms were combined to conduct the iterative process of marker ordering. The error correction strategy of SMOOTH^50^ was used according to parental contributions to genotypes, and a k-nearest neighbour algorithm was applied to impute missing genotypes. Skewed markers were added to this map by applying a multipoint method of maximum likelihood. Map distances were estimated using Kosambi’s mapping function.

### Genome synteny analysis

To determine genome conservation between zombi pea and other closely related legumes, the SNP markers used to construct the linkage map were subjected to a BLASTN search against reference genome sequences of cowpea [*V*. *unguiculata* (L.) Walp.]^30^, mungbean [*V*. *radiata* (L.) Wilczek]^26^, azuki bean [*V*. *angularis* (Ohwi) Ohwi and Ohashi]^31^, and common bean [*P*. *vulgaris* L.]^51^. The synteny levels between each zombi pea LG and the chromosomes of each legume species were illustrated using Circos v.67-7^52^. LGs of zombi pea were plotted using cM lengths, while chromosomes of each legume were plotted using physical lengths.

### Evaluation of seed resistance to *C*. *maculatus* and *C*. *chinensis*

A population of *C*. *chinensis* and a population of *C*. *maculatus* were reared on seeds of susceptible mungbean cultivar ‘Kamphaeng Saen 2’ and under controlled conditions in a room at 28 °C and 60% relative humidity. The evaluations of resistance to these insects were carried out following the procedures described by Somta *et al*.^38^, with minor modifications. Briefly, 40 seeds from each plant in the F_2_ population were placed in a plastic box. Then, 10 pairs (10 males and 10 females) of newly emerged adult insects were introduced into the box, allowed to lay eggs for 7 days and then removed. The infested seeds were maintained at 28 °C and 60% relative humidity. The numbers of seeds damaged by the bruchids [seeds with hole(s)] were counted at 30 days after insect introduction (DAI), and then counted every 5 days until 60 DAI. Each time, damaged seeds were removed from the boxes. The cumulative number of total seeds damaged by the bruchids at each counting dates was calculated and converted into percentage. Then, the percentage of damaged seeds was used to calculate the AUDPS^52^. AUDPS is an improve version of area under disease progress curve, which measures the progressive development of disease severity in plants. Thus, AUDPS is an indicator of the progression of the bruchid infestation severity in this study. The percentage of damaged seeds at 60 DAI and the AUDPS value of each F_2_ plants were used for data analyses.

### Heritability estimation for the resistance

The *H*^2^ values of PDS and AUDPS caused by each bruchid species in the F_2_ population were calculated using following formula:

$${H}^{2}=[{{\rm{\sigma }}}_{{\rm{F}}2}^{2}-(\frac{{{\rm{\sigma }}}_{{\rm{TVNu}}240{\rm{X}}}^{2}+{{\rm{\sigma }}}_{{\rm{TVNu}}1623{\rm{Y}}}^{2}}{2})]/{{\rm{\sigma }}}_{{\rm{F}}2}^{2},$$

where ${{\rm{\sigma }}}_{{\rm{F}}2}^{2},\,{\sigma }_{{\rm{TVNu}}240{\rm{X}}}^{2},\,{\rm{and}}\,{\sigma }_{{\rm{TVNu}}1623{\rm{Y}}}^{2}$ are variances of the F_2_ population, TVNu 240, and TVNu 1623, respectively.

### QTL analysis

The QTL analysis was conducted using inclusive composite interval mapping (ICIM) method^53^ implemented in QTL IciMapping 4.1 software^54^. The percentage of damaged seeds and AUDPS resulting from *C*. *chinensis* and *C*. *maculatus* infestations were used to locate QTLs associated with pest resistance. The ICIM was performed at every 0.1 cM using a probability in stepwise regression of 0.001. A significant LOD threshold for QTLs of each trait was determined using a 3,000 permutation test at *P* = 0.01.

## Supplementary information

Construction of a high density linkage map and genome dissection of bruchid resistance in zombi pea (Vigna vexillata (L.) A. Rich)
