# Supplementary material for: Construction of a high density linkage map and genome dissection of bruchid resistance in zombi pea (Vigna vexillata (L.) A. Rich)
Source: Sci Rep. 2019 Aug 12;9:11719. doi: 10.1038/s41598-019-48239-5 (PMC6690978; doi:10.1038/s41598-019-48239-5)
Supplement: Supplementary file 1 — Construction of a high density linkage map and genome dissection of bruchid resistance in zombi pea (Vigna vexillata (L.) A. Rich) [file 41598_2019_48239_MOESM1_ESM.pdf]

Supplementary Information

Construction of a high density linkage map and genome dissection of bruchid resistance in zombi pea (*Vigna vexillata* (L.) A. Rich)

Kitiya Amkul[1,2,3], Lixia Wang[4*], Prakit Somta[1,2,3*], Suhua Wang[4], Xuzhen Cheng[4]

[1]Department of Agronomy, Faculty of Agriculture at Kamphaeng Saen, Kasetsart University, Kamphaeng Saen Campus, Nakhon Pathom 73140, Thailand

[2]Center of Advanced Studies for Agriculture and Food, Kasetsart University Institute of Advanced Studies, Kasetsart University, Bangkok 10900, Thailand

[3]Center of Excellence on Agricultural Biotechnology: (AG-BIO/PERDO-CHE), Bangkok 10900, Thailand

[4]Institute of Crop Sciences, Chinese Academy of Agricultural Sciences, Beijing 100081, China

* Correspondence: Dr. Prakit Somta, Department of Agronomy, Faculty of Agriculture at Kamphaeng Saen, Kasetsart University, Kamphaeng Saen Campus, Nakhon Pathom 73140, Thailand. Email: agrpks@ku.ac.th, Dr. Lixia Wang, Institute of Crop Sciences, Chinese Academy of Agricultural Sciences, Beijing 100081, China. Email: wanglixia03@caas.cn

Table S1 Genome synteny between genetic linkage map of *Vigna vexillata* (LG1 – LG11) and reference genome sequence of cowpea (*V. unguiculata*), mungbean (*V. radiata*), azuki bean (*V. angualaris*), and common bean (*Phaseolus vulgaris*)

**LGs of zombi pea (*V. vexillata*)**	**Corresponding chromosomes of related legume species**			
	**Cowpea (*V. unguiculata*)**	**Mungbean (*V. radiata*)**	**Azuki bean (*V. angularis*)**	**Common bean (*P. vulgaris*)**
1	1	2, 3, 5	4, 7	1, 5
2	2	11	10	2, 3
3	3	7	1	2, 3
4	4	1	11	4
5	5	4, 5	4, 7	5, 8
6	6	10	5	6
7	7	8	8	7
8	8	6	3	1, 8
9	9	5	2	9
10	10	9	9	10
11	11	2	6	11

Table S2. Correlation between resistance-related traits caused by *C. chinensis* and *C. maculatus* in the F_2 population of the cross between cultivated zombi pea "TVNu 240" and wild zombi pea "TVNu 1623"

	**% damaged seeds caused by *C. chinensis***	**AUDPS caused by *C. chinensis***	**% damage seeds caused by *C. maculatus***
**AUDPS caused by *C. chinensis***	0.9990***		
**% damaged seeds caused by *C. maculatus***	0.7174***	0.6970***	
**ADUPS caused by *C. maculatus***	0.7217***	0.7044***	0.9968***

***Significant at $P < 0.001$
